# Screening and Functional Evaluation of Four *Larix kaempferi* Promoters

**DOI:** 10.3390/plants13192777

**Published:** 2024-10-03

**Authors:** Chen-Yi Zhang, Zha-Long Ye, Li-Wang Qi, Ling Yang, Wan-Feng Li

**Affiliations:** 1 State Key Laboratory of Tree Genetics and Breeding, College of Forestry, Northeast Forestry University, Harbin 150040, China; 19831803558@163.com; 2 State Key Laboratory of Tree Genetics and Breeding, Key Laboratory of Tree Breeding and Cultivation of the National Forestry and Grassland Administration, Research Institute of Forestry, Chinese Academy of Forestry, Beijing 100091, China; kemiye@caf.ac.cn (Z.-L.Y.); lwqi@caf.ac.cn (L.-W.Q.); 3 College of Forestry, Beijing Forestry University, Beijing 100083, China

**Keywords:** biotechnology, conifer, GUS, larch, transformation, UBQ

## Abstract

Promoters are powerful tools for breeding new varieties using transgenic technology. However, the low and unstable expression of target genes is still a limiting factor in *Larix kaempferi* (Lamb.) Carr (Japanese larch) genetic transformation. In this study, we analyzed *L. kaempferi* transcriptome data, screened out highly expressed genes, cloned their promoters, and constructed plant expression vectors containing the *β-glucuronidase* (*GUS*) reporter gene driven by these promoters. Recombinant vectors were introduced into the *L. kaempferi* embryogenic callus by means of the *Agrobacterium*-mediated transient or stable genetic transformation method, and the promoter activity was then determined by measuring *GUS* expression and its enzyme activity in the transformed materials. Four highly expressed genes were identified: *L. kaempferi Zhang Chen Yi-1* (*LaZCY-1*), *Zhang Chen Yi-2* (*LaZCY-2*), *Translationally Controlled Tumor Protein (LaTCTP)*, and *ubiquitin* (*LaUBQ*). The 2000 bp fragments upstream of ATG in these sequences were cloned as promoters and named *pLaZCY-1*, *pLaZCY-2*, *pLaTCTP*, and *pLaUBQ*. Semi-quantitative and quantitative RT-PCR analyses of transient genetic transformation materials showed that all four promoters could drive *GUS* expression, indicating that they have promoter activities. Semi-quantitative and quantitative RT-PCR analyses and the histochemical staining of stable genetic transformation materials showed that the *pLaUBQ* promoter had higher activity than the other three *L. kaempferi* promoters and the *CaMV35S* promoter. Thus, the *pLaUBQ* promoter was suggested to be used in larch genetic transformation.

## 1. Introduction

*Larix kaempferi* (Lamb.) Carr (Japanese larch) is an important tree for afforestation in China because it has a fast growth rate and wide adaptation [1,2]. Genetic transformation technology breaks the boundaries of conventional breeding, and it can be used to create new germplasm quickly for the directional improvement of forest trees [3]. *Agrobacterium*-mediated genetic transformation technology is a widely used research tool and has been used in the genetic improvement of larch [4,5,6,7,8,9,10,11,12].

The stable expression of target genes in transgenic plants is important for the genetic improvement of target traits, which largely depends on promoter activity [13]. The Cauliflower Mosaic virus 35S (*CaMV35S*) promoter is a constitutive promoter widely used in plant genetic transformation [11,14,15,16,17]; however, its activity is lower than that of native promoters in larch [18] and other conifers [19]. Therefore, identifying native and powerful promoters is of great importance.

For example, by using Chinese fir (*Cunninghamia lanceolata*) protoplast transient expression technology, the activities of Chinese fir *Cula11* and *Cula08* promoters were compared with that of the *CaM35S* promoter, and the results showed that *Cula11* and *Cula08* promoters have stronger activities [19]; notably, the *Cula11* promoter also has stronger activity than the *CaM35S* promoter in transgenic poplar [19]. By using *L. kaempferi* transient transformation technology, the activity of the promoter of a *L. kaempferi* native gene, *LaSCL6*, was compared with that of the *CaMV35S* promoter, and the results showed that the *LaSCL6* promoter has stronger activity [18]. These data show that screening native and efficient promoters from conifers for high-level constitutive gene expression is feasible and relevant.

Therefore, to identify powerful promoters that are specifically tailored to larch, we analyzed *L. kaempferi* transcriptome data, screened out four highly expressed genes, and compared their promoter activities in *L. kaempferi* transient and stable genetic transformation. This study aims to provide a potentially powerful tool for genetic transformation technology in larch.

## 2. Results and Discussion

### 2.1. Four Highly Expressed L. kaempferi Genes Were Screened, and Their Promoters Were Cloned and Analyzed

**Figure 1:**
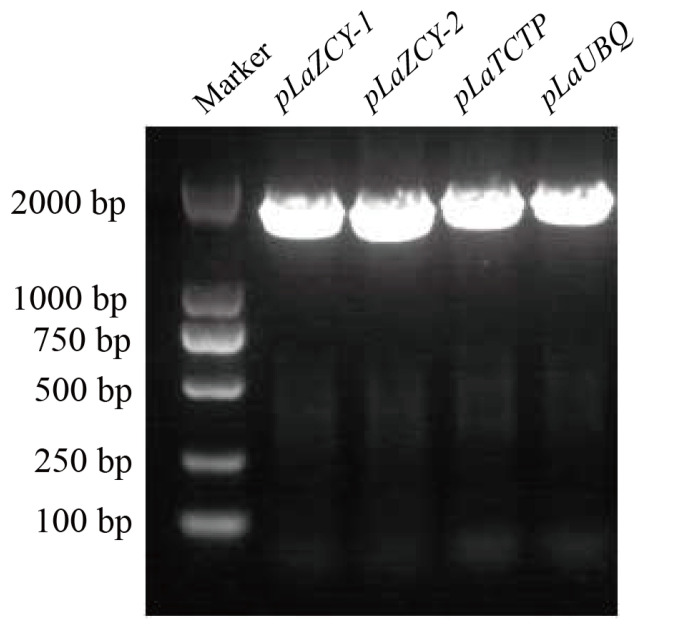
PCR amplification of *Larix kaempferi* promoters.

**Figure 2:**
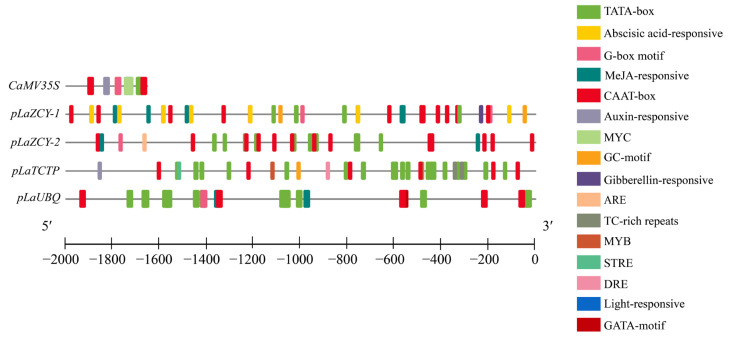
An analysis of the cis-acting elements of the five promoters.

Transcriptomic data provide a better understanding of the global regulation of gene expression [20]. Many *L. kaempferi* transcriptomes are currently available [21], making it possible and easy to screen highly expressed *L. kaempferi* genes. After analyzing three sets of transcriptome data (Table 1), four highly expressed genes were screened, and they were named *L. kaempferi Zhang Chen Yi-1* (*LaZCY-1*), *Zhang Chen Yi-2* (*LaZCY-2*)*, Translationally Controlled Tumor Protein (LaTCTP)*, and *ubiquitin* (*LaUBQ*). Among these genes, *LaZCY-1* and *LaZCY-2* had not been annotated (Table 2). Based on the source of the transcriptome, these four genes were highly expressed in *L. kaempferi*, indicating that their promoters have constitutive activities. Their promoter sequences were then cloned (Figure 1) and analyzed (Figure 2).

It was found that these four *L. kaempferi* promoters are composed of many TATA-boxes and CAAT-boxes (Figure 2), which are core promoter elements required for transcription initiation [22]. The TATA-box is the first core promoter motif that was discovered, as well as the best known core promoter element [23], and it enables the precise initiation of transcription [24]. The CAAT-box is a conserved promoter element and has the greatest influence on the transcription initiation frequency; the change in any base in this region will greatly affect the transcription intensity of the target gene [22,25]. In addition, other elements related to hormones, light, defense, and stress were also found in these four promoters (Figure 2). The existence of these core elements and other elements in these four genes’ promoters might lead to their high expression, showing their potential for use in larch genetic transformation.

**Table 1:** Screening of four highly expressed genes of *Larix Kaempferi*.

Rank	Different Seasons [21]	Different Ages [26,27]	Different Tissues or Organs [28,29]
1	LK_I_c24897_16125	LK_T_003052_c01_g01_i02.p1 *	LK_I_c20093_79657
2	LK_I_c29610_18909 *	LK_I_c13741_08564 *	LK_I_c26700_17377
3	LK_I_c03008_01536	LK_I_c29610_18909 *	LK_I_c13741_08564 *
4	LK_I_c20093_79657	LK_I_c15119_09293	LK_I_c34911_22534
5	LK_I_c13741_08564 *	LK_I_c24880_46025	LK_I_c35735_23103 *
6	LK_T_003052_c01_g01_i02.p1 *	LK_I_c35735_23103 *	LK_T_003052_c01_g01_i02.p1 *
7	LK_I_c15119_09293	LK_I_c08547_36770	LK_I_c08682_05299
8	LK_I_c17207_10752	LK_I_c05858_35168	LK_I_c29610_18909 *
9	LK_I_c26700_17377	LK_I_c40290_25505	LK_I_c06228_35388
10	LK_I_c35735_23103 *	LK_I_c32634_50059	LK_I_c00501_32008

* indicates the four common highly expressed genes in three transcriptomes.

**Table 2:** Annotation of four highly expressed genes in *Larix kaempferi*.

ID	Gene Name	Description
LK_I_c29610_18909	*LaZCY-1*	−
LK_I_c35735_23103	*LaZCY-2*	−
LK_I_c13741_08564	*LaTCTP*	Translationally Controlled Tumor Protein [30]
LK_T_003052_c01_g01_i02.p1	*LaUBQ*	Ubiquitin family

− means no annotation.

### 2.2. The Activities of the Four Promoters Were Detected in L. kaempferi Transient Transformation Based on GUS Expression

**Figure 3:**
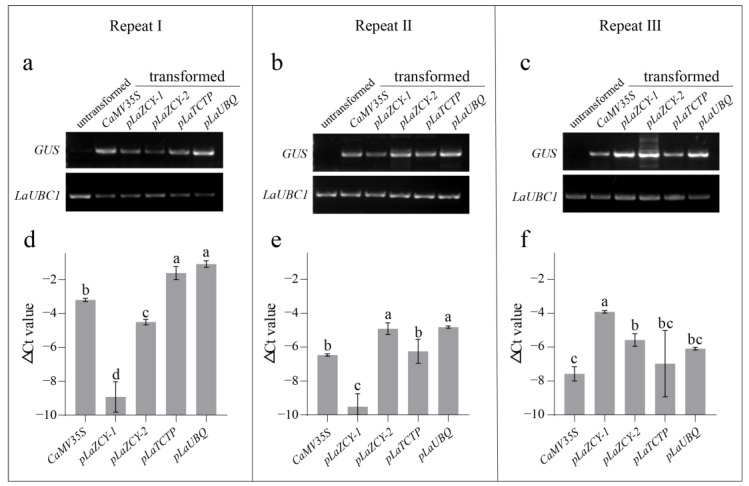
Semi-quantitative (**a**–**c**) and quantitative (**d**–**f**) RT-PCR analyses of *GUS* expression driven by five promoters separately in *Larix kaempferi* transient transformation. Error bars represent standard deviations of three replicates. Differences between each sample were analyzed using LSD, *p* ≤ 0.05, indicated by lowercase letters.

To verify the activities of these four *L. kaempferi* promoters, transient transformation was performed three times, and semi-quantitative and quantitative RT-PCRs were then performed to measure *GUS* expression. The semi-quantitative RT-PCR results showed that the amplified fragments of *GUS* were detected in all transformed *L. kaempferi* but not in the untransformed *L. kaempferi* (Figure 3a–c). The quantitative RT-PCR results showed that *GUS* was expressed in all transformed *L. kaempferi* at different levels (Figure 3d–f). These results indicated that *GUS* was successfully expressed in *L. kaempferi* transient transformation, and the four *L. kaempferi* promoters can drive *GUS* expression. However, transient transformation can only be utilized for temporary expression [31]; thus, *L. kaempferi* stable transformation was also carried out to further detect the activities of these four promoters.

### 2.3. GUS Expression and Activity Were Easily Detected in L. kaempferi Stable Transformation When Driven by pLaUBQ Promoter

**Figure 4:**
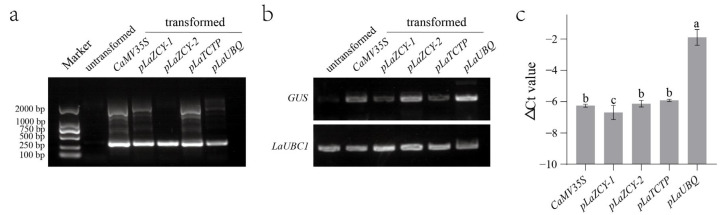
PCR (**a**), semi-quantitative (**b**), and quantitative (**c**) RT-PCR analyses of *GUS* in *Larix kaempferi* stable transformation. Error bars represent standard deviations of three replicates. Differences between each sample were analyzed using LSD, *p* ≤ 0.05, indicated by lowercase letters.

To verify the insertion of *GUS* into the *L. kaempferi* genome, polymerase chain reaction (PCR) amplification was performed with *GUS*-specific primers (Table S1) and with *L. kaempferi* DNA as a template. The results showed that the amplified fragments of *GUS* were detected in the transformed *L. kaempferi* but not in the untransformed *L. kaempferi* (Figure 4a). With the *L. kaempferi* cDNA as a template, semi-quantitative and quantitative RT-PCR were performed to measure *GUS* expression. The semi-quantitative RT-PCR results showed that the amplified fragments of *GUS* were detected in all transformed *L. kaempferi* but not in the untransformed *L. kaempferi* (Figure 4b). The quantitative RT-PCR results showed that *GUS* was expressed in all transformed *L. kaempferi*, and the highest level occurred when *GUS* expression was driven by the *pLaUBQ* promoter (Figure 4c). Thus, these results indicated that *GUS* was successfully integrated into the *L. kaempferi* genome and strongly expressed when driven by the *pLaUBQ* promoter.

**Figure 5:**
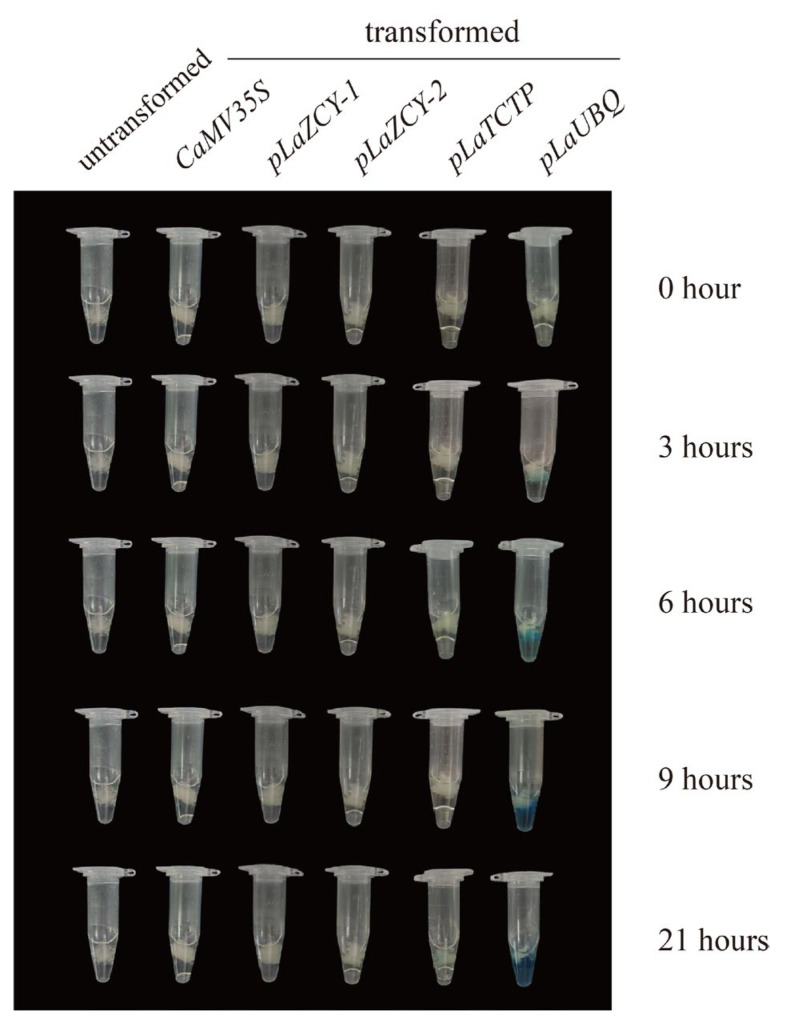
Pictures of *Larix kaempferi* transgenic callus after GUS staining for 0, 3, 6, 9, and 21 h.

GUS staining was also used to detect the activities of four *L. kaempferi* promoters in stable transformation. After three hours, a blue color was only observed in cultures transformed with vectors harboring the *pLaUBQ* promoter (Figure 5); after 21 h, it was also observed in cultures transformed with vectors harboring the *pLaTCTP* promoter (Figure 5), but it was darker in cultures transformed with vectors harboring the *pLaUBQ* promoter. Notably, a blue color was not observed in cultures transformed with vectors harboring the other three promoters after 21 h of staining (Figure 5). These results show that the *GUS* activities were different when they were driven by different promoters in *L. kaempferi* stable transformation, and the *pLaUBQ* promoter had the strongest activity.

### 2.4. pLaUBQ Promoter Can Be Used in L. kaempferi Transformation and Gene Editing Systems

In plant transformation systems, the *CaMV35S* promoter is extensively used to drive the constitutive expression of selection marker genes, reporter genes, and genes of interest [32,33,34]. However, the use of the *CaMV35S* promoter to drive gene expression in conifer transformation is not ideal [17]. In this study, we suggested that the *pLaUBQ* promoter can be used in *L. kaempferi* transformation to replace the *CaMV35S* promoter because the *pLaUBQ* promoter’s activity is higher than the other three *L. kaempferi* promoters and the *CaMV35S* promoter in stable *L. kaempferi* transformation.

*LaUBQ* belongs to the ubiquitin family. Ubiquitin is a highly conserved eukaryotic protein that is expressed to a considerable degree in plant tissues at different developmental stages [35,36]. In this study, *LaUBQ* was also found to be strongly expressed in *L. kaempferi*, showing its promoter’s stable and high activity. In fact, the ubiquitin promoter is always used and has good performance. For example, when the maize ubiquitin promoter *ZmUbi1* drives the expression of *aryloxyalkanoate dioxygenase*, it is more active than the *CaMV35S* promoter [37].

Notably, the ubiquitin promoter also shows great potential in CRISPR-mediated genome editing systems. For example, the use of the ubiquitin promoter *UBQ2* improves editing efficiency by increasing the expression of *Cas9* [38]. Targeted genome editing in *L. kaempferi* has been achieved using the CRISPR/Cas9 system, wherein the STU-Cas9 system driven by the larch *LarPE004* promoter (a sequence fragment upstream of the initiation codon of polyubiquitin) [39] (6.1–11.0%) or *ZmUbi* promoter (2.7–3.5%) has better editing efficiency at a single site than the STU-Cas9 system driven by *CaMV35S* (1.8–2.0%) [40]. Thus, the future application of the *pLaUBQ* promoter in *L. kaempferi* CRISPR systems may also improve the efficiencies of targeted mutagenesis.

In addition, the *pLaUBQ* promoter is a native promoter; it may not be easily modified after being transformed into the *L. kaempferi* genome, so its modification, which may lead to the transgene silencing [41], can be avoided. In this study, the *pLaUBQ* promoter sequence is 2000 bp, and a little long compared with the *CaMV35S* promoter sequence (346 bp). In the future, we will detect the activities of the *pLaUBQ* promoters of different lengths to identify the short and powerful promoter fragments that are easily used in *L. kaempferi* transformation, and will measure the transgene expression and stability in the regenerated *L. kaempferi* plants.

## 3. Materials and Methods

### 3.1. Re-Analyzing L. kaempferi Transcriptomes to Screen Highly Expressed Genes

To obtain highly expressed genes, three sets of published transcriptomes were re-analyzed, and they were produced from different *L. kaempferi* materials, including stems collected from different seasons (from February 2019 to February 2020) [21], ages (1-, 4-, 8-, 12-, 20-, and 50-year-old dormant *L. kaempferi* trees; 1-, 2-, 5-, 10-, 25-, and 50-year-old active *L. kaempferi* trees) [26,27], and different tissues or organs (callus, roots, phloem, and leaves) [28,29]. In each set of data, the top ten highly expressed genes were obtained based on the rank of the minimum expression level of each gene, and the common genes in three sets of data were considered to be highly expressed genes (Table 1).

### 3.2. Promoter Cloning, Sequence Analysis, and Vector Construction

**Figure 6:**
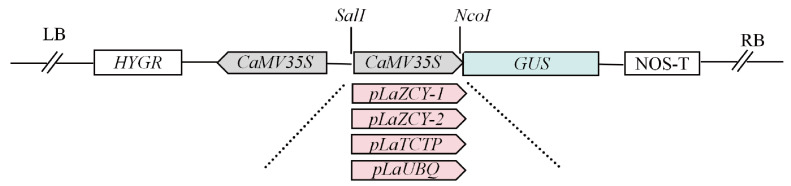
A schematic representation of the vectors used for *Larix kaempferi* genetic transformation.

The 2000 bp fragment upstream of the initiation codon of the highly expressed gene was obtained from the genomic data [42], and after cloning, the cis-acting components in promoters were predicted using PlantCARE (https://bioinformatics.psb.ugent.be/webtools/plantcare/html/, accessed on 1 March 2024). The *CaMV35S* promoter in the binary vector pCAMBIA1301 was replaced by cloned promoter fragments with *SalⅠ* and *NcoⅠ* restriction enzymes using the homologous recombination method, and there was a selection marker gene hygromycin phosphotransferase (*HYGR*) and a reporter gene, *GUS,* in the recombinant vectors (Figure 6), which were used for genetic transformation.

### 3.3. Agrobacterium-Mediated L. kaempferi Transient and Stable Transformation

The *L. kaempferi* embryogenic cell line C6 was used for genetic transformation; it was induced from immature embryos [43] and subsubstituted every 15 days on a solid proliferation medium [8] in the dark (25 ± 2 °C). The transformation was performed as described earlier [10], with some modifications. The *Agrobacterium tumefaciens* strain GV3101, harboring the recombinant vector, was cultured at 28 °C for 48 h on a solid Luria–Bertani (LB) medium supplemented with 50 mg/L kanamycin and 50 mg/L rifampicin. A single Agrobacterium clone was selected and cultured in 2 mL of liquid LB medium supplemented with the same antibiotics at 28 °C for 12 h on a rotary shaker (200 rpm). The culture was transferred to 50 mL of LB medium and grown under the same conditions for 12 h to OD_600_ = 0.6–0.8. The bacterial cells were collected via centrifugation at 4000 rpm for 10 min.

The pellet was re-suspended to OD_600_ = 0.05 and cultured in 100 mL of liquid proliferation medium, and 100 µM acetosyringone was added. Approximately 10 g (~50 mL) of the embryogenic cultures was added to an equal volume of the bacterial suspension. A vacuum filter was used to remove excess liquid from the cultures. The filtered cultures were spread on a solid proliferation medium supplemented with 100 µM acetosyringone and co-cultivated for 24 h in the dark at 25 °C, and then the cultures were sampled as transient transformation materials. The transient genetic transformation was repeated three times.

When the stable genetic transformation was performed, the pellet was re-suspended to OD_600_ = 0.1. After co-cultivation for 48 h in the dark at 25 °C, the cultures were washed four times each with 100 mL sterile distilled water and 100 mL cefotaxime solution (500 mg/L), and then they were transferred to a solid proliferation medium supplemented with 5 mg/L hygromycin and 500 mg/L cefotaxime. After ~4–8 weeks, hygromycin-resistant cultures were transferred to the same medium for further selection. The surviving cultures were maintained on the solid proliferation medium with 5 mg/L hygromycin until they were sampled as stable transformation materials.

### 3.4. Nucleic Acid Extraction and cDNA Synthesis

DNA was extracted with the Plant GenomicDNA Kit (TIANGEN, Beijing, China) from the untransformed callus for promoter cloning and from both the untransformed and transformed materials for PCR analysis.

Total RNA was extracted from both the untransformed and transformed callus with the EasyPure RNA Kit (TransGen Biotech, Beijing, China) according to the manufacturer’s protocol for gene expression analysis. Then, a 2.5 µg aliquot of total RNA was reverse-transcribed into cDNA with TransScript One-Step gDNA Removal and cDNA Synthesis SuperMix (TransGen Biotech, Beijing, China).

### 3.5. PCR, Semi-Quantitative RT-PCR, and Quantitative RT-PCR (qRT-PCR)

The PCR amplification of the sequence fragment of *GUS* (337 bp) was conducted to verify the insertion of *GUS* into the *L. kaempferi* genome with *L. kaempferi* DNA as a template. The following PCR program was used: 1 cycle at 94 °C for 2 min, then 35 cycles at 98 °C for 10 s, 58 °C for 5 s, and 68 °C for 5 s, followed by 1 cycle at 68 °C for 2 min. The PCR products were analyzed via electrophoresis in 1% agarose gel.

The PCR amplification of the sequence fragment of *GUS* (337 bp) was conducted to measure *GUS* expression with *L. kaempferi* cDNA as a template and *LaUBC1* (ON887160) [44,45] as the internal control. The same PCR program as above was used, in addition to 30 cycles. The PCR products were analyzed via electrophoresis in 1% agarose gel.

The qRT-PCR was conducted with a Bio-Rad CFX96 PCR system using TB Green^®^ Premix ExTaq™ (Tli RNase H Plus) (Takara, Shiga, Japan). Each reaction was carried out with 0.5 µM of gene-specific primers (Table S1) and 2 µL of a diluted cDNA sample in a total reaction system of 25 µL. *LaUBC1* was used as the internal control. Four technical replicates were used for each sample, the ∆CT value (CT*_LaUBC1_* − CT*_GUS_*) is used to represent the results, and the data are presented as mean ± SD values.

Excel was used for data statistics and analysis, and GraphPad Prism was used for drawing. The significance of the differences was analyzed with Statistical Product and Service Solutions (SPSS Statistics 26, IBM Corp., New York, NY, USA) software with an analysis of variance (ANOVA).

### 3.6. GUS Staining

Almost the same amounts of stable transformation materials were used for GUS staining, which was performed with a β-Galactosidase Reporter Gene Staining Kit (Huayueyang, Beijing, China) in a 1.5 mL centrifuge tube according to the kit instructions. After incubation at 37 °C in the dark for 3, 6, 9, or 21 h, the materials were photographed.

## 4. Conclusions

In this study, four highly expressed *L. kaempferi* genes were screened through transcriptome analysis, and their promoter activities were measured. All four promoters demonstrated activity because they could drive *GUS* expression. Among these promoters, the *pLaUBQ* promoter could replace *CaMV35S* for use in *L. kaempferi* genetic transformation and gene editing systems because it had a higher level of activity than the other three *L. kaempferi* promoters and the *CaMV35S* promoter.

## Data Availability

The data presented in this study are available upon reasonable request from the corresponding author.

## Supplementary Materials

The following supporting information can be downloaded at: https://www.mdpi.com/article/10.3390/plants13192777/s1, Table S1: Primer sequences used in this study.
