# Supplementary material for: Screening and Functional Evaluation of Four Larix kaempferi Promoters
Source: Plants (Basel). 2024 Oct 3;13(19):2777. doi: 10.3390/plants13192777 (PMC11478676; doi:10.3390/plants13192777)
Supplement: Supplementary file 1 [file plants-13-02777-s001.zip › plants-3187763-supplementary.pdf]

Table S1. Primer sequences used in this study.

Gene name	Sequence (5′–3′)	Application
LaZCY-1	Forward-GTGACTGGCTTTGCTGTTGTTGGATTTGG	PCR
	Reverse-AAATCCAAGAATTTCACCTATGACAATG	
	Forward-tacccggggatcctctagagGTGACTGGCTTTGCTGTTGTTGGATTTGG	Vector construction
	Reverse-ttaccctcagatctaccatgAAATCCAAGAATTTCACCTATGACAATG	
LaZCY-2	Forward-GGTGTAGAATTTGCTGGCCAATGTTCTGATGG	PCR
	Reverse-CCAGGACAGATTGGAAATGGTTATGCACCC	
	Forward-tacccggggatcctctagagGGTGTAGAATTTGCTGGCCAATGTTCTGATGG	Vector construction
	Reverse-ttaccctcagatctaccatgCCAGGACAGATTGGAAATGGTTATGCACCC	
LaTCTP	Forward-GTCATCAAAGCATAACTAGGAGTCC	PCR
	Reverse-CTGAGCAAGTCCTGGTAGACG	
	Forward-tacccggggatcctctagagGTCATCAAAGCATAACTAGGAGTCC	Vector construction
	Reverse-ttaccctcagatctaccatgCTGAGCAAGTCCTGGTAGACG	
LaUBQ	Forward-AACTTATTCCTACCAAAATCTAAAAATTAC	PCR
	Reverse-CTTGAGACCGGAAAATAACTAGGGATATACC	
	Forward-tacccggggatcctctagagAACTTATTCCTACCAAAATCTAAAAATTAC	Vector construction
	Reverse-ttaccctcagatctaccatgCTTGAGACCGGAAAATAACTAGGGATATACC	
GUS	Forward-CGACGCTCACACCGATACCATC	Semi-quantitative and quantitative RT-PCR
	Reverse-GCACACTGATACTCTTCACTCCACAT	
LaUBC1	Forward-TGGCGTCCAAAAGGATTCTCA	
	Reverse-TCCCATGATTGTAGCTTGCCA	
